# Supplementary material for: Shared Genetic Architecture Between Endometriosis and Psychiatric Conditions May Explain Comorbidity
Source: medRxiv. 2025 Oct 24:2025.10.22.25338556. Preprint. [Version 1] doi: 10.1101/2025.10.22.25338556 (PMC12633614; doi:10.1101/2025.10.22.25338556)
Supplement: Supplement 7 [file media-7.docx]

## STable 10 FUMA results

| #Genomic risk loci | 193 |
| --- | --- |
| #lead SNPs | 248 |
| #Ind. Sig. SNPs | 606 |
| #candidate SNPs | 35482 |
| #candidate GWAS tagged SNPs | 19530 |
| #mapped genes | 527 |
